# Unusual Anatomic Variant of the Biliary Tree

**DOI:** 10.7759/cureus.47206

**Published:** 2023-10-17

**Authors:** Tatiana Fernandez Trokhimtchouk, Luis F Flores, Álvaro Morillo Cox, Estefanie S Otanez, Jose R Negrete, Andrés V Ayala

**Affiliations:** 1 General Surgery, Universidad Internacional del Ecuador, Quito, ECU

**Keywords:** magnetic resonance cholangiopancreatography, hepatic ducts, cholelithiasis, laparoscopic cholecystectomy, biliary tract

## Abstract

Accurate identification of anatomical variations in the biliary tree is crucial in hepatobiliary surgeries, including the widely performed laparoscopic cholecystectomy. Coexisting anomalies, though rare, present challenges for surgeons. This case study follows a 43-year-old female post-sleeve gastrectomy, diagnosed with mild gallstone pancreatitis and choledocholithiasis, who underwent early cholecystectomy. Intraoperatively, a 3 mm aberrant right hepatic duct and three 1 mm subvesicular ducts were identified. Recognizing these variants is pivotal for surgical success. Utilizing preoperative imaging, intraoperative vigilance, and advanced techniques like cholangiography are essential in preventing complications. Continuous education and collaborative experiences among surgical teams are integral in enhancing patient safety in these complex procedures.

## Introduction

Variations in the anatomy of the biliary tree have long been recognized as critical considerations in surgical interventions involving the hepatobiliary system. The intricate network of bile ducts, including aberrant configurations and accessory ducts, poses a formidable challenge for surgeons. Accurate identification and thorough understanding of these anatomical nuances are imperative to prevent inadvertent bile duct injury.

While anatomical variations within the biliary tree are not uncommon, the coexistence of multiple anomalies in a single patient is an exceedingly rare clinical scenario [1]. Among these variations, the presence of Luschka ducts, also known as subvesical ducts, alongside an aberrant right hepatic duct, represents a particularly infrequent occurrence [2]. This unique combination presents a surgical conundrum and is an important risk factor for biliary surgical lesions during cholecystectomy [3].

In this case presentation, we outline the clinical course of a 43-year-old female, one year post-sleeve gastrectomy, who presented with symptoms indicative of acute mild gallstone pancreatitis with concomitant choledocholithiasis. Following the resolution of this condition through endoscopic retrograde cholangiopancreatography (ERCP), a subsequent surgical exploration was undertaken for gallbladder removal. During this procedure, an anatomical anomaly was discovered, consisting of a 3 mm aberrant right hepatic duct draining into the cystic duct, accompanied by three 1 mm subvesicular ducts connecting to the common hepatic duct. A thorough understanding of anatomical variations is essential for assessing the practicality of surgical procedures and executing surgeries in this area without complications [3].

A substantial body of literature is dedicated to preventing bile duct injuries, with numerous guidelines published due to the significant morbidity associated with these complications. Anatomical variants stand out as one of the primary risk factors for inadvertent bile duct injury. Therefore, contributing to the existing body of knowledge is paramount in raising awareness and facilitating adaptability during surgical interventions [4].

## Case presentation

A 43-year-old female, one year post-sleeve gastrectomy, presented to the emergency department with a 30-hour history of colicky epigastric pain radiating to the back, accompanied by nausea but no vomiting. Initial examination revealed normal vital signs and tenderness on the mesogastrium, epigastrium, and right upper quadrant, along with a positive Murphy’s sign.

**Figure 1:**
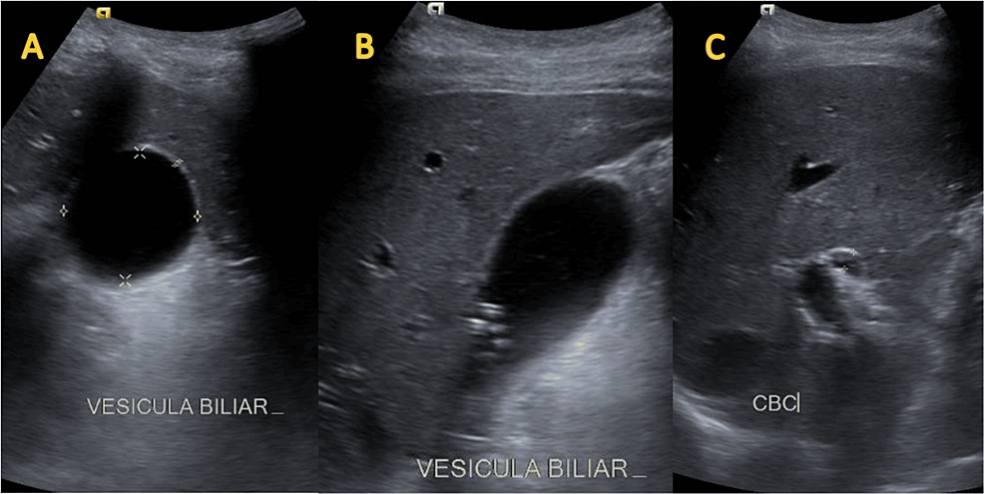
Ultrasound of the upper abdomen shows a gallbladder with 2 mm walls (A) containing multiple stones (B) and a 6 mm common bile duct (C)

Laboratory findings demonstrated no leukocytosis, no neutrophilia, signs of cholestasis, elevated pancreatic enzymes, and a C-reactive protein of 52 mg/dL. Imaging revealed cholecystolithiasis without evidence of cholecystitis on upper abdominal ultrasound and a 6 mm common bile duct diameter (Figure 1).

**Figure 2:**
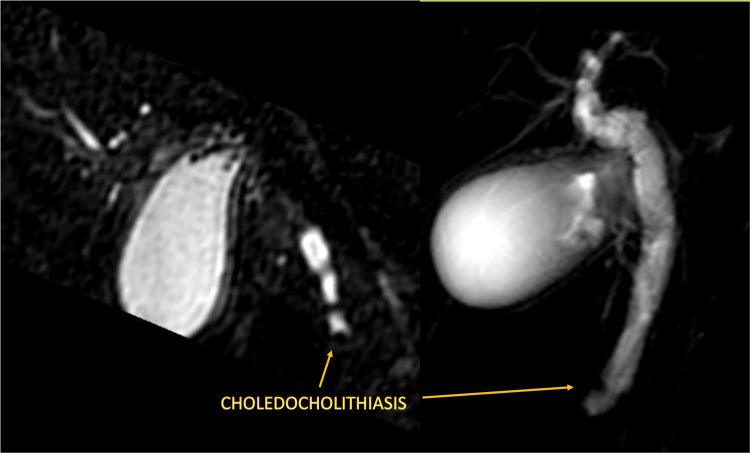
MRCP confirming choledocholithiasis MRCP: magnetic resonance cholangiopancreatography

In accordance with the American Society for Gastrointestinal Endoscopy (ASGE) 2019 guidelines, an intermediate risk of choledocholithiasis was established. The diagnosis was confirmed by magnetic resonance cholangiopancreatography (MRCP) as shown in Figure 2. Subsequently, the patient underwent an ERCP, successfully extracting biliary sludge and performing a papillotomy without complications.

**Figure 3:**
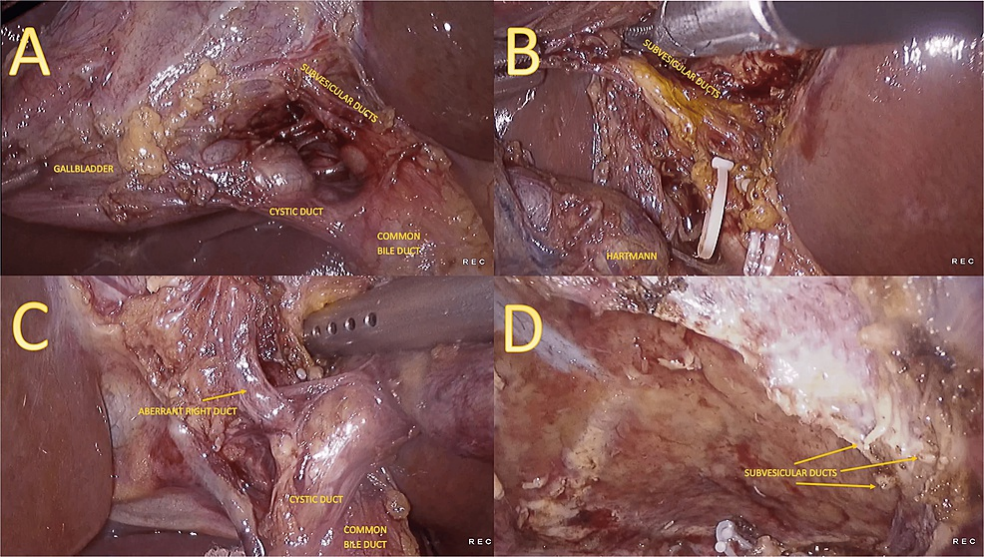
Intraoperative findings Panel A shows subvesicular ducts, found during the initial dissection of Calot’s triangle, which drain into the common bile duct. In panel B, bile coming from these ducts is seen. The right aberrant duct inserted into the cystic duct is seen in panel C. Panel D shows the subvesicular ducts after completion of the gallbladder ectomy.

During laparoscopic cholecystectomy, surgical exploration revealed variant biliary anatomy (Figure 3). Specifically, a 3 mm aberrant right hepatic duct was identified as draining into the cystic duct, along with three 1 mm subvesicular ducts from the cystic plate connecting to the common hepatic duct. Preservation of the aberrant duct was achieved by clipping the cystic duct prior to its insertion while Luschka ducts were ligated with non-absorbable clips. A drain was left in situ.

**Figure 4:**
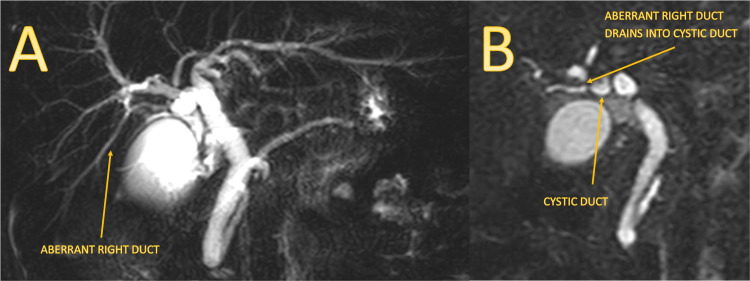
MRCP shows the aberrant right duct (A), which drains into the cystic duct (B). Subvesicular ducts could not be identified. MRCP: magnetic resonance cholangiopancreatography

Postoperatively, the patient experienced an uneventful recovery. The surgical drain was removed on day 5 with minimal serous output. Pathology indicated chronic cholecystitis within an acute episode. A retrospective revision of MRCP affirmed the variant anatomy observed during surgery (Figure 4).

## Discussion

The development of the biliary tree is a complex process with origins in the endodermal tissue of the foregut. During embryogenesis, the hepatic diverticulum gives rise to the intrahepatic bile ducts while the extrahepatic ducts develop from the hepatic diverticulum's caudal portion. Aberrant right hepatic ducts and Luschka ducts arise from developmental irregularities in this process. The aberrant right hepatic duct is a result of an anomalous connection between the right hepatic duct and cystic duct while Luschka ducts, often considered accessory ducts, are remnants of embryonic bile ducts that persist in the adult anatomy [5].

The right aberrant duct is a rare anatomical variant where the right hepatic duct follows an abnormal course, often connecting directly to the cystic duct. Its incidence is reported to be around 5-10% of cases, making it a relatively uncommon but significant anatomical variation. In clinical practice, this variant can pose challenges during surgical interventions, particularly cholecystectomy and hepatectomy procedures. Failure to recognize the presence of a right aberrant duct can lead to inadvertent injury, emphasizing the criticality of preoperative imaging and intraoperative awareness [6].

Luschka ducts, or subvesicular ducts, represent an additional anatomical variation within the biliary tree. These are small bile ducts that communicate between the liver parenchyma and the gallbladder bed, bypassing the main biliary tree. Their presence is relatively common, with reported incidence varying widely from 12% to 50% [7]. While often asymptomatic, Luschka ducts can have clinical significance, particularly in cases of biliary injury during surgical procedures involving the gallbladder. These ducts can mimic bile leaks, leading to postoperative complications.

Both the right aberrant duct and Luschka ducts significantly contribute to the risk of iatrogenic bile duct injury during surgery [8]. In the case of the right aberrant duct, its abnormal course and connection with the cystic duct can be mistaken for normal anatomy, potentially resulting in unintended ductal injury. Similarly, Luschka ducts, if not identified, may be mistaken for small vessels or bile ducts, leading to inadvertent injury. To mitigate these risks, meticulous surgical planning, advanced imaging techniques, and intraoperative cholangiography can play pivotal roles in preventing such complications.

Literature on cases involving both right aberrant ducts and Luschka ducts is limited. A notable case reported by Aoki et al. underscores the importance of cholangiography during surgery to identify these anatomical variants [2]. The scarcity of reported cases highlights the need for heightened awareness and scrutiny when reviewing preoperative imaging for potential anatomical anomalies.

The "critical view of safety," a well-documented concept in the literature, encompasses three essential components. One crucial aspect involves liberating the lower third of the liver bed from the gallbladder. This criterion holds particular significance in discerning aberrant anatomy and safeguarding against inadvertent injuries [9].

In this case, both preoperative MRCP and cholangiography from the ERCP were meticulously reviewed to validate the intraoperative findings. The patient's uneventful recovery and the minimal serous drainage further affirm the effective management.

## Conclusions

This case serves as a pertinent reminder of the criticality of recognizing and understanding anatomical variants within the biliary tree. Awareness of the presence of aberrant right hepatic ducts and Luschka ducts is paramount in preventing iatrogenic bile duct injury. Meticulous preoperative assessment, including advanced imaging techniques, and intraoperative vigilance can significantly enhance patient safety in surgical interventions involving the hepatobiliary system. Continuous education and sharing of experiences are essential in advancing the field and improving patient outcomes.
